# A collision tumor composed of colon adenocarcinoma and large cell neuroendocrine carcinoma with gingival metastasis: a case report

**DOI:** 10.3389/fonc.2025.1633770

**Published:** 2025-08-06

**Authors:** Zhiqiang Zhang, Junfeng Zhang, Peng Chen, Xuexia Kou, Yunsheng Lan, Yingdong Jia

**Affiliations:** ^1^ Department of Clinical Medical College, North Sichuan Medical College, Nanchong, Sichuan, China; ^2^ Department of Gastrointestinal Surgery, Suining Central Hospital, Suining, Sichuan, China

**Keywords:** colon adenocarcinoma, large cell neuroendocrine carcinoma, collision tumor, gingival metastasis, neuroendocrine transformation

## Abstract

Collision tumors represent a relatively uncommon form of tumor manifestation, characterized by the independent coexistence of multiple distinct types of tumors. Large cell neuroendocrine carcinoma of the colorectum is a rare entity, with limited reports describing collision tumors involving adenocarcinoma and large cell neuroendocrine carcinoma. Distant metastases of colorectal cancer typically occur in sites such as bone, liver, and lungs; however, metastasis to the gingiva is rarely encountered. This paper reports a case of a collision tumor comprising colonic adenocarcinoma and large cell neuroendocrine carcinoma, with accompanying gingival metastasis.

## 1 Case description

Colorectal cancer accounts for 18% of malignant tumors (1). The vast majority of them are adenocarcinomas. Large cell neuroendocrine carcinoma is extremely rare in the large intestine, accounting for only 0.1 - 0.6% of colorectal cancers (2). There have been few reports on colonic collision tumors with the coexistence of adenocarcinoma and large cell neuroendocrine carcinoma (3). The common metastatic sites of colorectal cancer mainly include the liver, lungs, bones, etc. Gingival metastasis is extremely rare in clinical practice (4). In this study, we report a unique case of a patient with ascending colon adenocarcinoma. Two years after surgical treatment, recurrence and multiple metastases occurred in the patient. Pathological biopsy of the gingival mass confirmed it as a metastasis of colon cancer. Subsequently, a palliative resection of the cancer lesion was carried out. A nodule was found beside the cancer lesion, and the pathological results showed that it was large cell neuroendocrine carcinoma. Through immunohistochemical and pathological examinations, it was diagnosed as a collision tumor with the coexistence of adenocarcinoma and large cell neuroendocrine carcinoma. In this paper, combined with relevant literature, an in-depth discussion is carried out on the diagnosis, treatment, and characteristics of this case.

A 45 - year - old female was diagnosed with ascending colon cancer in 2022. She then underwent radical right hemicolectomy. The postoperative pathological stage was pT4N0M0. Following the surgery, the patient underwent regular chemotherapy. The initial treatment regimen consisted of capecitabine in combination with oxaliplatin. This therapeutic approach primarily achieves anti-tumor effects through multi-target inhibition of tumor cell proliferation and induction of apoptosis. However, due to notable toxicities and adverse effects observed during the treatment, including bone marrow suppression and gastrointestinal disturbances, the patient elected to discontinue further chemotherapy after completing two cycles.

In March 2023, a recurrence of abdominal wall tumor was identified and determined to represent a solitary lesion. Following complete local resection, pathological examination confirmed the diagnosis of adenocarcinoma. Postoperative treatment was adjusted to a combination regimen of mFOLFOX6 (oxaliplatin + 5-fluorouracil + leucovorin) and bevacizumab. This therapeutic strategy primarily exerts anti-tumor effects through the inhibition of tumor cell proliferation and angiogenesis; however, it is associated with a relatively high risk of bone marrow suppression and hemorrhage. In May 2024, local recurrence at the primary site was observed. During subsequent ongoing therapy, progressive metastases to the liver, lung, and pelvis emerged, indicating disease progression. Consequently, the treatment strategy was modified to include immunotherapy with camrelizumab combined with radiotherapy. Camrelizumab is capable of blocking the PD-1/PD-L1 immune inhibitory pathway, thereby activating and enhancing T cell cytotoxicity. Between July and November 2024, significant tumor progression was noted, including an increase in both the number and size of metastatic lesions, as well as the appearance of new rib metastases. Palliative local radiotherapy was administered. During the course of further treatment, the patient gradually developed hematochezia, with an increasing volume of blood loss over time.

Between July and November 2024, the tumor demonstrated significant progression, characterized by an increase in the number and size of metastatic foci, along with the emergence of new rib metastases. Local radiotherapy was subsequently administered. During the subsequent treatment, the patient gradually experienced hematochezia, with the amount gradually increasing. In November 2024, severe hematochezia led to severe anemia, prompting the patient to undergo palliative resection of the primary lesion once again.

**Figure 1:**
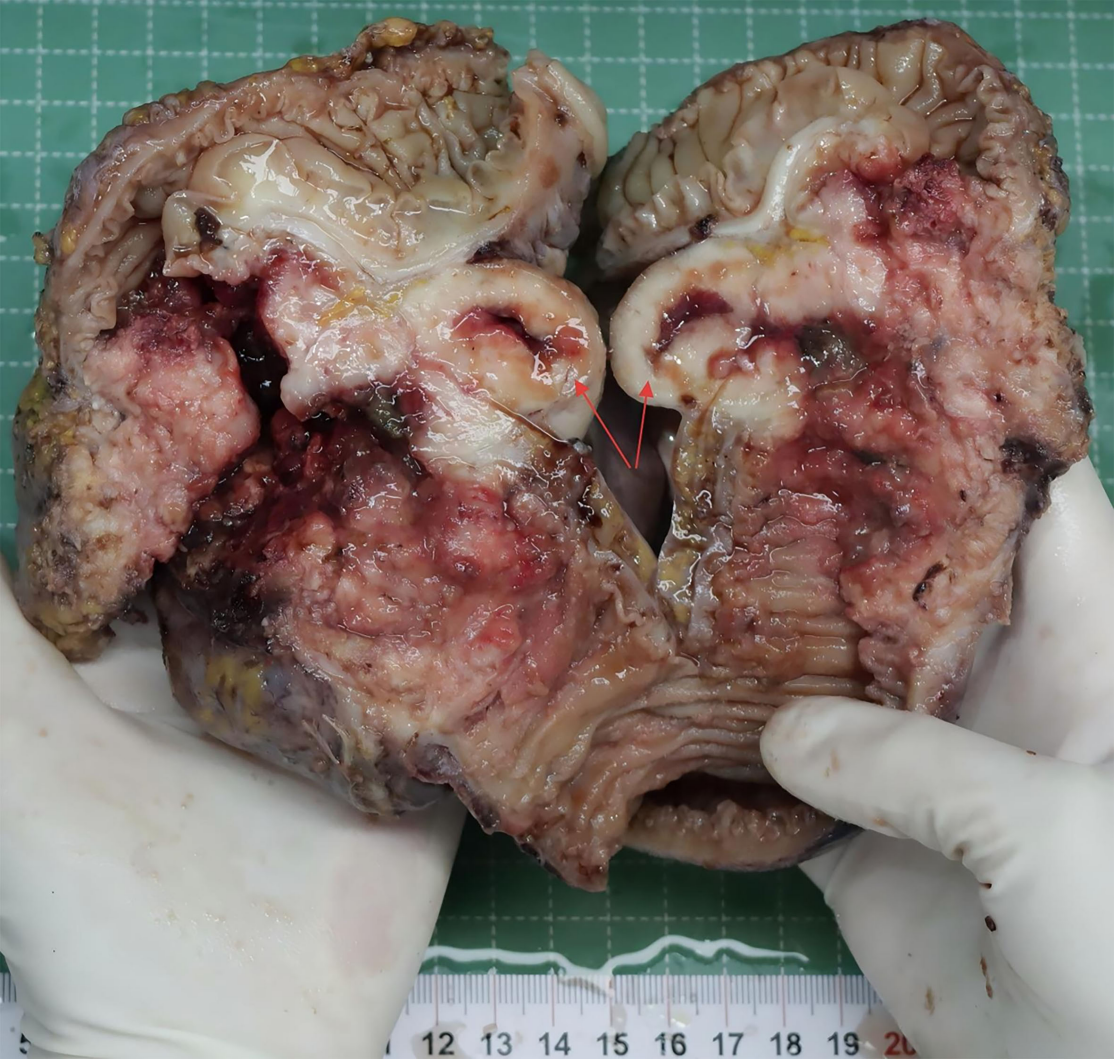
The resected colon tumor (the red arrow indicates the nodules of large cell neuroendocrine carcinoma).

**Figure 2:**
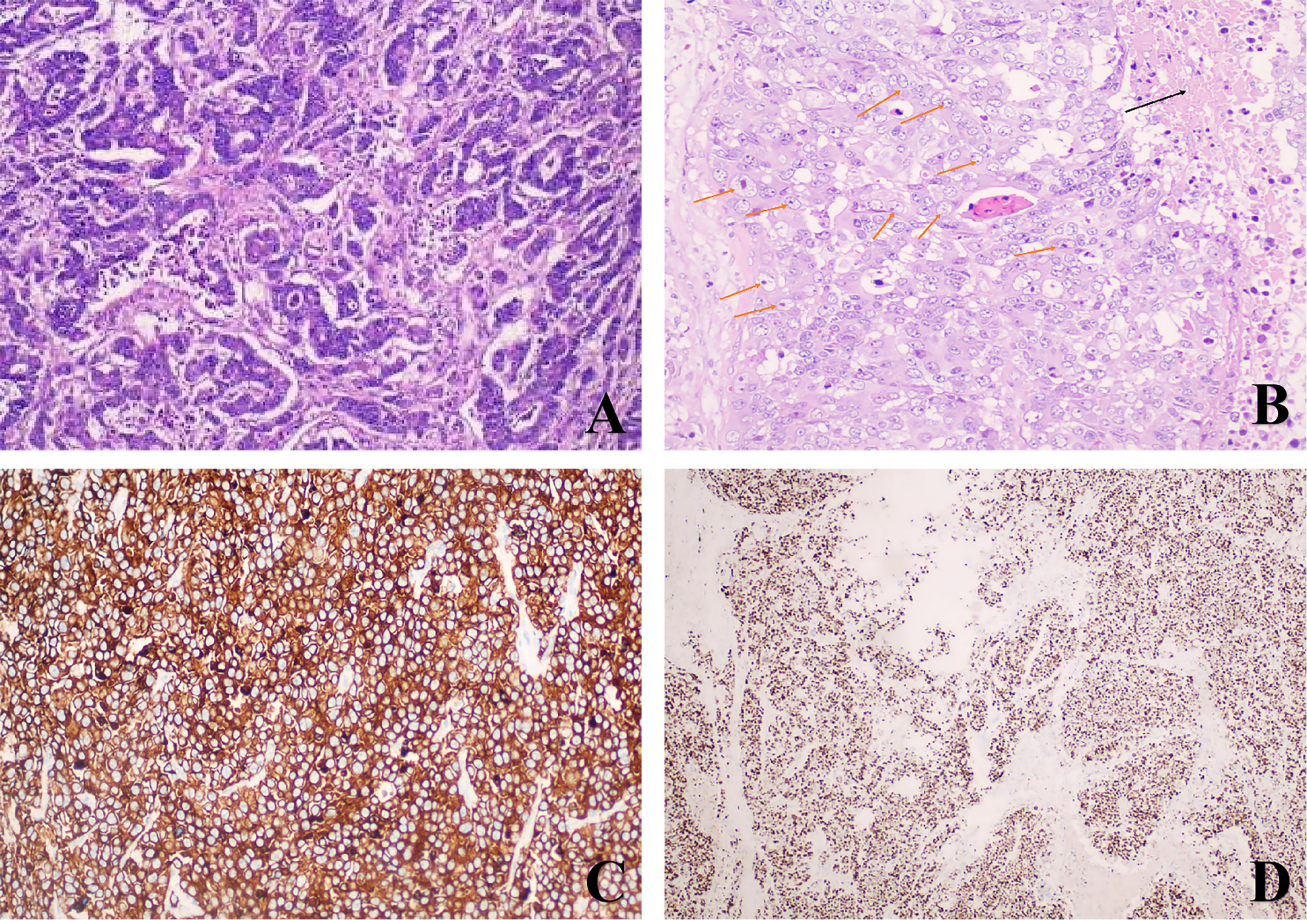
Histological and immunohistochemical examination: **(A)** Adenocarcinoma components of the colon (Hematoxylin and eosin, ×100); **(B)**Large atypical cells and necrotic foci (Hematoxylin and eosin, ×200)(The black arrows indicate the necrotic areas; The orange arrows indicate some large cell neuroendocrine cells); **(C)** Tumor cells show strong and diffuse positivity for Synaptophysin (EnVision, ×200); **(D)** The Ki67 proliferation index was 90% (EnVision, ×100).

The postoperative pathology revealed ulcerative moderately - to - poorly differentiated adenocarcinoma, which had invaded the surrounding tissues. Notably, in the mesenteric region, adjacent to the tumor, there was a solid nodule (**Figure 1**). Pathological examination of this nodule showed large cells with large, vesicular nuclei and prominent nucleoli. Immunohistochemical analysis indicated Syn(+), Ki67(+, 90%), which was consistent with the diagnosis of large cell neuroendocrine carcinoma (**Figure 2**). The two tumor components existed independently and did not invade each other. Morphologically, this finding was consistent with the diagnosis of a collision tumor composed of colon adenocarcinoma and large cell neuroendocrine carcinoma.

**Figure 3:**
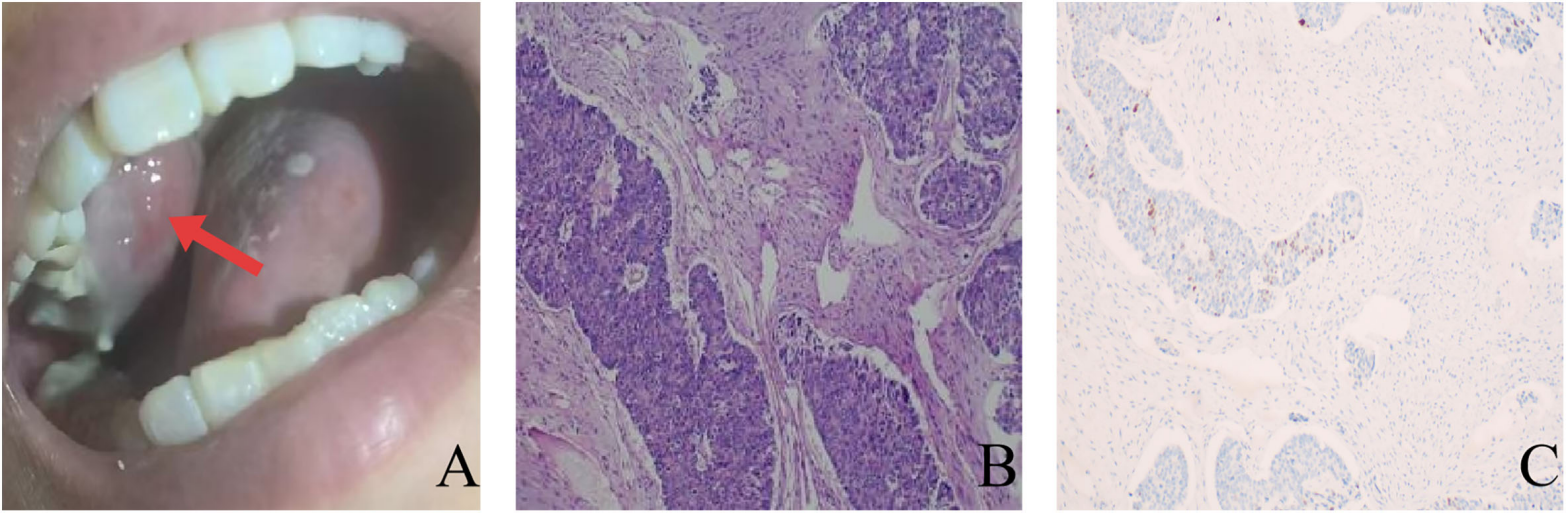
**(A)** Exophytic mass on the right upper gingiva (The red arrow indicates a gingival mass.); **(B)** Pathological examination revealed adenocarcinoma components (Hematoxylin and eosin, ×100); **(C)** Immunohistochemistry indicates positive CDX2 (EnVision, ×100).

Furthermore, a mass was observed on the patient’s right upper gingiva, which gradually enlarged. Biopsy of this mass was conducted, and immunohistochemical results indicated that it was consistent with adenocarcinoma, with CDX2(+) (**Figure 3**). Considering its origin, it was diagnosed as gingival metastasis of colon cancer.

## 2 Discussion

Colon adenocarcinoma is a prevalent malignancy; however, the occurrence of collision tumors in this context is exceedingly rare (5). A collision tumor refers to a morphological entity where two or more distinct tumor components coexist independently without intermixing (6). In this report, we describe a case of a collision tumor comprising advanced colon adenocarcinoma and large cell neuroendocrine carcinoma, with an unusual gingival metastasis. This type of colorectal adenocarcinoma with a neuroendocrine component typically lacks the characteristic neuroendocrine symptoms and is commonly diagnosed through postoperative histopathological examination (7). Clinically, it is crucial to differentiate such collision tumors from mixed neuroendocrine-non-neuroendocrine neoplasms (MiNENs), composite tumors, and other related entities. The diagnostic criteria for MiNENs require the simultaneous presence of both neuroendocrine and non-neuroendocrine components, each constituting at least 30% of the tumor mass (8). In the present case, the neuroendocrine component accounts for only approximately 10% of the tumor, failing to meet the diagnostic threshold for MiNENs. Composite tumors, conversely, are characterized by the intermingling of two distinct tumor components. In our case, the two tumor components are spatially segregated with well-defined histological boundaries, thus fulfilling the diagnostic criteria for collision tumors (6). Currently, there is no definitive consensus regarding the origin of collision tumors. Within the academic community, three theoretical hypotheses have gained broad acceptance (1): the two types of tumor cells originate from distinct cell lineages (2); they arise from a common multipotent stem cell; and (3) the coexistence of the two independent tumors is coincidental (5, 9). Regarding the origin of the large cell neuroendocrine carcinoma in this particular case, considering the patient’s medical history, it is hypothesized that it arose through neuroendocrine differentiation.

Neuroendocrine differentiation (NED) has been demonstrated to occur in adenocarcinomas of various non-endocrine organs. For instance, a study revealed that neoadjuvant treatment in rectal adenocarcinoma enhanced the endocrine phenotype of tumor cells (8). In investigations involving other tumor types, it has been observed that transformed neuroendocrine carcinomas often emerge in lung adenocarcinomas treated with anti-EGFR therapy and prostate adenocarcinomas subjected to androgen deprivation therapy. These findings suggest that the selective pressure imposed by anti-tumor therapies may induce neuroendocrine differentiation (10, 11). Numerous studies have established a robust correlation between neuroendocrine differentiation in colorectal cancer and an unfavorable prognosis for patients. In the present case, after initial platinum-based chemotherapy and targeted therapy, the tumor exhibited rapid and significant progression, leading to a cascade of complications. Additionally, components of large cell neuroendocrine carcinoma were identified as independently existing within subsequent tumor lesions. Based on the patient’s treatment course, it is hypothesized that the neuroendocrine phenotype observed in this case may have arisen from cytotoxic injury caused by radiotherapy and chemotherapy. This injury could have triggered a phenotypic transformation of tumor cells toward a neuroendocrine lineage (8).This phenotypic transformation may confer metastatic colorectal cancer with resistance to anti-angiogenic agents and immune checkpoint inhibitors, ultimately reducing treatment efficacy.

Oral metastatic malignancies are exceedingly rare, accounting for less than 1% of all oral malignancies (12). The mandible is the predominant site of metastasis, whereas gingival involvement is relatively uncommon (13). Studies have shown that in males, oral metastatic tumors predominantly originate from lung cancer, renal cell carcinoma, and prostate cancer. Conversely, in females, oral metastatic malignancies are primarily linked to breast cancer and renal cell carcinoma (14). Metastases from colorectal cancer to the oral cavity are comparatively rare.

Colorectal cancer predominantly metastasizes to organs such as the bones, lungs, and liver (15). Metastasis to supraclavicular regions is relatively uncommon, and oral metastasis is particularly rare. When it does occur in the oral cavity, the mandible is most frequently affected (16). This phenomenon may be associated with the Batson venous plexus, a valveless paravertebral venous system that connects the pelvic cavity, abdominal cavity, and thoracic cavity (17). When colorectal cancer invades the abdominal wall vasculature, it creates anatomical conditions that facilitate distant hematogenous metastasis of cancer cells. Moreover, oral metastasis is often accompanied by metastasis to other organs (14). In a small subset of patients, oral symptoms may present as the primary clinical manifestation (18). Therefore, accurately identifying the primary origin of oral tumors and their metastatic spread to other organs is critical for effective patient diagnosis and treatment. Numerous studies have demonstrated that the implementation of perioperative surgical management strategies for patients with oral metastatic tumors can, to some extent, prolong patient survival and improve their quality of life (16).

Regarding treatment, due to the rarity of such cases, there is currently no well-established oncological treatment protocol available to guide the management of this type of condition. Nonetheless, the selection of chemotherapy regimens can be based on the predominant components. In this case, palliative surgery was performed for symptom relief. Regrettably, the patient declined further treatment.

## 3 Conclusion

Herein, we report a rare case of a collision tumor comprising colon adenocarcinoma and large cell neuroendocrine carcinoma, with gingival metastasis. Further documentation of similar cases is crucial for enhancing our understanding of the biological behavior, therapeutic options, and overall prognosis of such tumors.

## Data Availability

The original contributions presented in the study are included in the article/supplementary material. Further inquiries can be directed to the corresponding authors.
